# *Strongyloides stercoralis* prevalence and diagnostics in Vientiane, Lao People’s Democratic Republic

**DOI:** 10.1186/s40249-020-00750-y

**Published:** 2020-09-21

**Authors:** Somaphone Chankongsin, Rahel Wampfler, Marie-Therese Ruf, Peter Odermatt, Hanspeter Marti, Beatrice Nickel, Valy Keoluangkhot, Andreas Neumayr

**Affiliations:** 1 Infectious Diseases Ward, Mahosot Hospital, Vientiane, Lao People’s Democratic Republic; 2 University of Basel, Basel, Switzerland; 3 Department of Medicine, Swiss Tropical and Public Health Institute, Basel, Switzerland; 4 Public Health and Tropical Medicine, College of Public Health, Medical and Veterinary Sciences, James Cook University, Townsville, Australia

**Keywords:** *Strongyloides stercoralis*, Strongyloidiasis, Wet smear, Baermann method, Koga agar plate culture, Real time detection PCR

## Abstract

**Background:**

Despite the high prevalence of strongyloidiasis in the Laotian population, Laotian hospitals still lack diagnostic capacity to appropriately diagnose *Strongyloides stercoralis* infections. This cross-sectional hospital-based study was conducted to assess the prevalence of *Strongyloides stercoralis* infection among hospitalized patients treated at Mahosot Hospital, the primary reference hospital of Lao People’s Democratic Republic (Lao PDR), and to validate feasible methods for diagnosing *S. stercoralis* infection at hospital’s laboratory.

**Methods:**

Between September and December 2018, stool samples of 104 inpatients were investigated for *S. stercoralis* infection by wet smear, Baermann technique, Koga Agar plate culture (KAPC), and real-time detection polymerase chain reaction (RTD-PCR) at the Infectious Diseases Ward of the Mahosot Hospital in Vientiane. The sensitivity, the specificity, the negative predictive value (NPV) of each diagnostic test, as well as their combination(s) was calculated using a composite reference standard (CRS). The correlation of the different test methods was assessed by chi-square or Fisher’s exact test. Cohen’s kappa coefficient was used to assess the diagnostic agreement of the different test methods.

**Results:**

The overall prevalence of *S. stercoralis* infections among the study population was 33.4%. The cumulative infection prevalence statistically significantly increased from the lowest age group of 40 years and below (22.4%), to the medium (40.0%) and to the oldest age group of 61 year and above (72.7%)(*P* = 0.003). The cumulative infection prevalence of CRS was considerably higher in male (40.4%) compared to female patients (28.1%), but not statistically different (*P* = 0.184). The diagnostic sensitivity of Baermann technique, KAPC, RTD-PCR, and the combination of Baermann technique and KAPC were 60.0, 60.0, 74.3, and 77.1%, respectively. Only 13 patients (37.1%) of the total 35 *S. stercoralis* patients diagnosed with any technique had a simultaneously positive diagnostic test with Baermann, KAPC and RTD-PCR.

**Conclusions:**

We identified Baermann technique and KAPC to be currently the most feasible and implementable standard methods for diagnosing *S. stercoralis* at a hospital setting such as Mahosot Hospital and provincial and district hospitals in Lao PDR and other low- and middle income countries in Southeast Asia.

**Trial registration:**

This study was approved by the National Ethics Committee for Health Research in Lao PDR (reference no. 083/NECHR) and by the Ethics Committee Northwest and Central Switzerland (reference no. 2018–00594).

## Background

Strongyloidiasis is a soil-transmitted helminth infection that affects, according to largely varying estimates, between 30 million and 370 million people worldwide [1, 2]. The two unique features of the helminth infection are the ability to cause autoinfection leading to persisting infection of its host and the free-living, non-parasitic life cycle in the environment.

Chronic strongyloidiasis in immunocompetent individuals is mostly asymptomatic or only causes unspecific non-acute symptoms related to the gastro-intestinal tract [3], which hampers early diagnosis and treatment. In immunocompromised patients however, the parasite’s ability to cause autoinfection may lead to hyperinfection with large numbers of invasive larvae finally causing fatal disseminated infection [4].

Although various diagnostic methods have been developed to diagnose *Strongyloides stercoralis* infection, the irregular shedding of larvae in stool of infected individuals as well as the limited sensitivity of the available diagnostic methods remains problematic [5, 6]. Thus, prevalence data from epidemiological studies are largely underestimating the true prevalence burden and patients remain undiagnosed or their diagnosis is delayed.

Information on the prevalence of infection with *S. stercoralis* varies considerably depending on the methodology used. In a review of available data until 2014, Schär and colleagues estimated the overall prevalence rates of *S. stercoralis* infection in South East Asia as follow: Cambodia: 23.6–25.6%; Indonesia: 4.2–6.3%; Lao PDR: 28.7–33.0%; Malaysia: 28.0–44.4%; Thailand: 26.0–28.2% [7]. In recent effort to model global *S. stercoralis* prevalence, an *S. stercoralis* infection prevalence between 10 and 15% has been estimated in Southeast Asian countries [2]. But a nation-wide survey in Cambodia detected a overall prevalence of 30.5% in the rural population [8].

The existing study data on *S. stercoralis* prevalence in Lao PDR indicate prevalence rates from 1.4% in Khammouan and Blikhamxay Provinces [9] to 44.2% in Xayaburi Province [10]. Of note, all studies previously reported were community-based investigations and all except three studies used an inadequate diagnostic approach. Furthermore, the reported prevalence rates were low, e.g. between 1.4% and 10.3%, for studies using an inadequate diagnostic approach consisting of either Kato-Katz (KK) or formalin-ether concentration technique (FECT) or both techniques [9, 11–13]. Much higher prevalence rates of more than 40% were reported when Baermann and or Koga agar plate culture (KAPC) techniques were applied [10, 14].

The larval output of *S. stercoralis* in stool is much lower than the egg output of other soil-transmitted helminths. Thus, microscopy-based direct methods, like simple wet smear preparation or Kato-Katz (a technique primarily used for the quantification of parasite eggs in epidemiological studies), and even conventional concentration techniques, like FECT (used to enhance the sensitivity for detecting protozoan cysts/oocysts and helminth eggs), are largely insufficient to diagnose *S. stercoralis* infection when only investigating a single sample. Concentration techniques specifically designed for the detection of *S. stercoralis*, such as Baermann or KAPC, provide more reliable results, but are cumbersome and therefore rarely used [5, 15]. Given the widespread use of insensitive diagnostic methods in curative health services in Lao PDR and other resource-poor countries in Southeast Asia, the true prevalence of *S. stercoralis* infection and hence, untreated patients, is unknown.

The objective of our study was to assess the prevalence of *S. stercoralis* infection among hospitalized patients at Mahosot Hospital, as an example of a curative health service in Lao PDR, and to validate different diagnostic methods at the hospital’s laboratory.

## Methods

### Study design and study area

This cross-sectional hospital-based study was conducted between September and December 2018 at the Infectious Diseases Ward of the Mahosot Hospital, Vientiane, Lao PDR. The majority of patients in Mahosot hospital originate from two provinces: Vientiane Prefecture (820 000 population) and Vientiane Province (420 000 population). Only four of the total 20 districts are considered urban (about 400 000 population). Rice subsistence farming is the main activity of households in the rural districts.

Based on the available data on *S. stercoralis* infection prevalence in Lao PDR we anticipated a prevalence rate of 40% and calculated the sample size, with a 95% confidence interval and a 10% margin of error, to be 92 patients.

### Study population

One hundred four consecutive patients ≥18 years of age admitted to the Infectious Diseases Ward of the Mahosot Hospital were (irrespective the cause of admission) enrolled in the study after consenting to participate.

### Laboratory methods

Stool samples were investigated by (1) wet smear, (2) Baermann technique, (3) KAPC, and (4) real-time detection polymerase chain reaction (RTD-PCR). One stool sample per participant was collected and material was split for the different methods accordingly. Each sample was investigated by each method once. Wet smear, Baerman, and KAPC were performed at the Mahosot hospital in Lao PDR immediately after arrival of stool samples, while RTD-PCR was performed in Basel, Switzerland.

#### Wet smear

A fresh stool sample of 5 g was mixed with 3 ml of 0.9% sodium chloride. A drop of the resulting dilution was put on a glass slide, covered with a coverslip, and microscopically examined for the presence of *S. stercoralis* larvae. This is the method routinely carried out at the laboratory of Mahosot Hospital for diagnosis of *S. stercoralis*.

#### Baermann technique

A fresh stool sample of 5 g was placed on gauze inserted into a glass funnel of 10 cm in diameter connected to a tube, and covered with tap water. The funnel was illuminated with a lamp from below and left for two hours at room temperature. The liquid in the tube containing the larvae was then transferred to a 15 ml tube and centrifuged for 5 min at ~ 344 g. The supernatant was discarded and the sediment microscopically examined for the presence of *S. stercoralis* larvae.

#### KAPC

Koga agar (KA) plates were prepared as described elsewhere [16]. A fresh stool sample of 2 g was placed in the center of a KA-plate and the closed Petri dish was then left to incubate for 48 h at room temperature. Afterwards, the plate was rinsed with 10% neutral buffered formalin solution, the eluent was centrifuged, and the sediment microscopically examined for the presence of *S. stercoralis* larvae. To avoid confusion of *Strongyloides* with hookworm larvae at microscopic examination, all larvae found were assessed for morphological characteristics (i.e., size of buccal cavity, presence of genital primordium [L1], presence of forked tail-end [L3]).

#### PCR

RTD-PCR testing was performed on stool samples of 100 mg conserved in 90% ethanol and stored in 1.5 ml cryo tubes at ambient temperature. For isolation of nucleic acids from stool samples the QIAamp DNA mini kit (Qiagen, Germany) was used according to an optimized protocol from Barda and colleagues [17]. The RTD-PCR targets the generic 28SrRNA gene of *Strongyloides* spp. as previously described on a fluorescence energy transfer (FRET)-based RTD-PCR format [18]. Primer and probe sequences were newly designed and adapted to TaqMan probe-based RTD-PCR format (forward primer: 5′-GCG AAC AAG TAC TGT GAA GGA AAA TTG-3′, reverse primer: 5′-TGG CTC TGT ATG CTT CCA TCG T-3′, and TaqMan probe: 5′-Yakima YellowACG TCC TCT TTA ACT CTC TCT CCG GBHQ1–3′), resulting in an amplicon of 92 basepairs (bp). The PCR reaction mixture contained 5 μl DNA, 1 x TaqMan Gene Expression Master Mix (Thermo Fisher Scientific, USA), 800 nmol forward and reverse primer, and 200 nmol probe in a total reaction volume of 25 μl. Thermal cycling started with a step at 50 °C for 2 min and 95 °C for 10 min, followed by 45 cycles of 95 °C for 15 s and 58 °C for 1 min. A plasmid containing the 92 bp amplicon sequence, as well as adjacent base pairs, was used as positive control. The assay was optimized on the plasmid and on characterized DNA from a *S. stercoralis* drug efficacy trial study [17, 19] (data not shown). Analytical sensitivity of the assay was one plasmid copy/μl DNA. Specificity was tested against DNA samples from *Blastocystis hominis, Chilomastix mesnili, Cryptosporidium hominis/parvum, Cyclospora cayetanensis, Cystoisospora belli, Dientamoeba fragilis, Encephalitozoon hellen, Entamoeba coli, E. dispar, E. hartmanni, E. histolytica, E. moshkovskii, Giardia lamblia, Hymenolepis nana, Iodamoeba bütschlii, Schistosoma mansoni, Taenia* spp*., Trichostrongylus* spp*.,* and was found to be 100%.

### Statistical analysis

The sensitivity, the specificity, the negative predictive value (NPV) of each diagnostic test, as well as their combination(s) was calculated using a composite reference standard (CRS). This CRS was defined as showing a positive result in any of the tests (Baermann, KAPC, or RTD-PCR). The correlation of the different test methods was assessed by chi-square test (*χ*^2^) or Fisher’s exact test, as appropriate, using Stata version 15 software (StataCorp LLC; Texas, USA). Cohen’s kappa coefficient was used to assess the diagnostic agreement of the different test methods (interpretation ranges: 0.01–0.20 = poor agreement; 0.21–0.40 = fair agreement; 0.41–0.60 = moderate agreement; 0.61–0.80 = substantial agreement; and 0.81–0.99 = strong agreement) with calculation of the 95% confidence interval (*CI*) and standard error (SE). For all statistical test of correlation, a *P*-value < 0.05 was considered significant.

## Results

### Demographic characteristics

In total, 154 consecutively identified patients were enrolled in the study of whom 104 patients (67.5%) provided sufficient stool sample to perform all testing and hence were retained in the analysis. The sociodemographic and medical characteristics of these patients are shown in Table 1. Slightly more women (54.8%) than men (45.2%) were in the study. Most patients (55.8%) had an age between 18 and 40 years.

**Table 1:** Sociodemographic and medical characteristics of the study participants

Patient characteristics		Number of patients (***n*** = 104)	Percentage (%)
**Age (years)**	18–40	58	55.8
	41–60	35	33.7
	61–86	11	10.6
**Gender**	Male	47	45.2
	Female	57	54.8
**Residency**	Urban	96	92.3
	Rural	8	7.7
**Admission diagnosis**	Dengue fever	42	40.4
	Pneumonia	13	12.5
	Septicemia	9	8.6
	Rickettsial disease	9	8.6
	Melioidosis	8	7.8
	Others/unspecified	23	22.1

### Diagnostic results

**Fig. 1:**
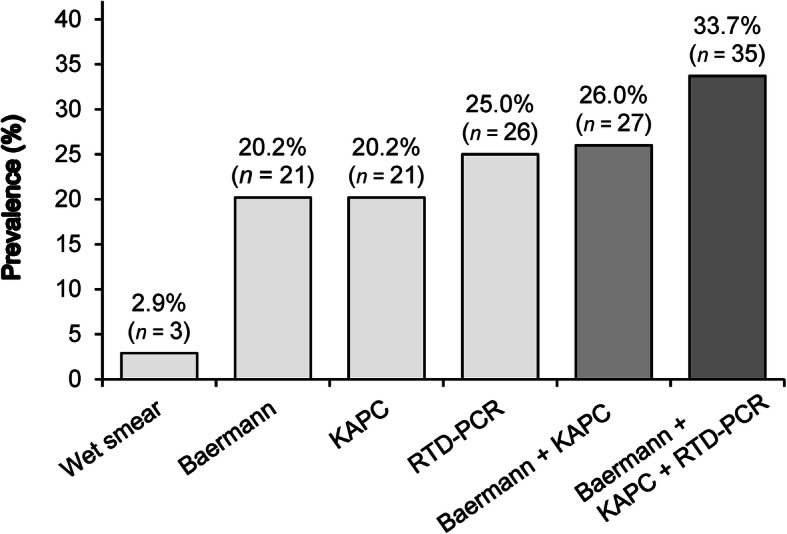
Prevalence of *Strongyloides stercoralis* infection according to the testing method(s) used. Baermann: Baermann concentration technique; KAPC: Koga agar plate culture; RTD-PCR: Real-time detection polymerase chain reaction; Colours. Light grey, Single test applied; Grey, Cumulative prevalence of two tests; Dark grey, Cumulative prevalence of three tests combined

The prevalence of *S. stercoralis* infection according to the testing method(s) used is depicted in Fig. 1. The *S. stercoralis* prevalence ranged from 2.9 to 25.5% when wet smear and RTD-PCR diagnostic test was applied as a single diagnostic test, respectively. We used the combined results of the Baermann, KAPC and RTD-PCR diagnostic test as composite reference standard (CRS). The cumulative infection prevalence for the CRS was 33.7%. In wet smear and Baermann, an infection with *Opisthorchis viverrini* was found in 4.8% (*n* = 5) and infection with hookworm was detected in 2.9% (*n* = 3) of the patients.

The cumulative infection prevalence of CRS was considerably higher in male (40.4%) compared to female patients (28.1%) but not statistically different (*P* = 0.184). The cumulative infection prevalence statistically significantly increased (*P* = 0.003) from the lowest age group of 40 years and below (22.4%), to the medium (40.0%) and to the oldest age group of 61 year and above (72.7%). The mean age of *S. stercoralis* infected patients was 40.4 years (SE = 2.7 years) and was statistically significantly higher (*P* = 0.004) than the age of *S. stercoralis* uninfected patients (36.8 years, SE = 1.8 years). The cumulative infection prevalence was 32.3% in patients from urban area and lower compared to patients from rural areas (50.0%, *P* = 0.308).

**Fig. 2:**
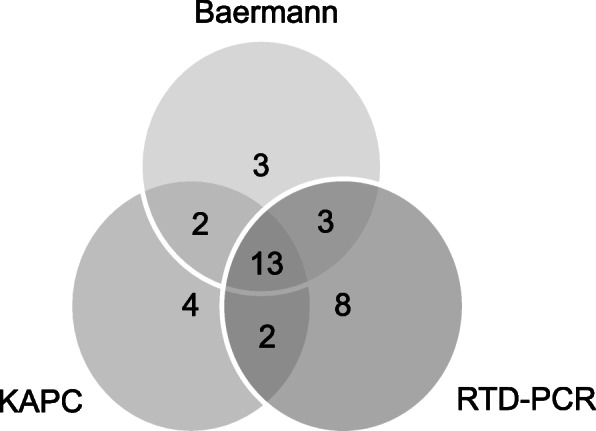
Positive test results for *Strongyloides stercoralis* grouped by diagnostic method. Baermann: Baermann concentration technique; KAPC: Koga agar plate culture; RTD-PCR: Real-time detection polymerase chain reaction

Only 13 patients (37.1%) of the total 35 *S. stercoralis* patients diagnosed with any technique had a simultaneously positive diagnostic test with Baermann, KAPC and RTD-PCR (Fig. 2). Each test identified *S. stercoralis* positive patients that were not identified with the other two diagnostic tests.

### Performance of diagnostic methods

The sensitivity, the specificity, and the negative predictive value (NPV) of the different diagnostic methods to detect *S. stercoralis* when compared against the CRS are summarized in Table 2. The sensitivity of the Baermann and KAPC tests were both 60.0%. RTD-PCR had a higher sensitivity of 74.3% and it was similar to the combined results of Baermann and KAPC tests (77.1%).

**Table 2:** Sensitivity, specificity, and NPV of the evaluated tests compared to the composite reference standard (CRS)

Diagnostic test		CRS	
		Positive	Negative
**Wet smear**	Positive	3	0
	Negative	32	69
	Sensitivity	8.6%	95% *CI*: 1.8–23.1%
	Specificity	100%	95% *CI*: 94.8–100%
	NPV	68.3%	95% *CI*: 58.3–77.2%
**Baermann**	Positive	21	0
	Negative	14	69
	Sensitivity	60.0%	95% *CI*: 42.1–76.1%
	Specificity	100%	95% *CI*: 94.8–100%
	NPV	83.1%	95% *CI*: 73.3–90.5%
**KAPC**	Positive	21	0
	Negative	14	69
	Sensitivity	60.0%	95% *CI*: 42.1–76.1%
	Specificity	100%	95% *CI*: 94.8–100%
	NPV	83.1%	95% *CI*: 73.3–90.5%
**RTD-PCR**	Positive	26	0
	Negative	9	69
	Sensitivity	74.3%	95% *CI*: 56.7–87.5%
	Specificity	100%	95% *CI*: 94.8–100%
	NPV	88.5%	95% *CI*: 79.2–94.6%
**Baermann + KAPC**	Positive	27	0
	Negative	8	69
	Sensitivity	77.1%	95% *CI*: 59.9–89.6%
	Specificity	100%	95% *CI*: 94.8–100%
	NPV	89.6%	95% *CI*: 80.6–95.4%

*Baermann* Baermann concentration technique, *CI* Confidence interval, *CRS* Composite reference standard, *KAPC* Koga agar plate culture, *RTD-PCR* Real-time detection polymerase chain reaction, *NPV* Negative predictive value

The diagnostic agreement of the different diagnostic tests according to Cohen’s kappa (κ) coefficient is shown in Table 3. We observed a substantial agreement of the diagnostic test results between Baermann and KAPC (**κ** = 0.642). The diagnostic agreements between Baermann and RTD-PCR (**κ** = 0.589) and KAPC and RTD-PCR (**κ** = 0.534) were moderate.

**Table 3:** Diagnostic agreement of diagnostic tests by Cohen’s kappa (κ) coefficient

Diagnostic methods	κ coefficient	SE	95% ***CI***	***P***-value
Baermann and KAPC	0.642	0.0981	0.456–0.828	0.0001
Baermann and RTD-PCR	0.589	0.0971	0.404–0.774	0.0001
KAPC and RTD-PCR	0.534	0.0971	0.341–0.727	0.0001

*SE* Standard error, *CI* Confidence interval, *Baermann* Baermann concentration technique, *KAPC* Koga agar plate culture, *RTD-PCR* Real-time detection polymerase chain reaction

Interpretation of Cohen’s kappa coefficient: 0.01–0.20 poor agreement, 0.21–0.40 fair agreement, 0.41–0.60 moderate agreement, 0.61–0.80 substantial agreement, 0.81–0.99 strong agreement

## Discussion

With a cumulative prevalence 33.7%, our data demonstrate a high prevalence of *S. stercoralis* infection among hospitalized patients in a semi-urban setting in Lao PDR. Previous work in a rural district of Southern Lao PDR showed a similar, though slightly higher prevalence of 41.0% [14]. However, since even at tertiary reference hospitals in Lao PDR diagnostic testing for *S. stercoralis* infection currently remains restricted to the highly insensitive direct microscopy of wet smear preparations, the identification of infected individuals remains highly problematic and the true infection burden underestimated in Lao PDR and many low- and middle income settings in Southeast Asia.

Since currently the most sensitive diagnostic methods to detect *S. stercoralis* in stool samples are Baermann, KAPC, and PCR, we chose these three techniques as CRS for our study. We want to note that our study is the first one to include PCR for determining the prevalence of *S. stercoralis* infection in Laotian patients. Although the implementation of PCR in routine diagnostic at Mahosot Hospital and other provincial and tertiary hospitals is currently not feasible, we nevertheless included PCR in our study to ensure evaluating Baermann and KAPC against the best possible CRS and to evaluate the potential future diagnostic value of PCR in this setting.

Our results further confirm and highlight that microscopy of wet smear preparations is largely insufficient to diagnose *S. stercoralis* infections (Fig. 1) and that this method needs to be replaced by more sensitive diagnostic methods in daily clinical practice.

Although we found Baermann and KAPC to have an equal sensitivity of 60%, Fig. 2 highlights that the two methods did not always identify the same infected individuals, which explains their additive value (Fig. 1) and sensitivity (77.1%; Table 2) when performed in parallel. Provided their availability, this finding supports the approach of parallel testing with both methods in clinical practice.

Although adding RTD-PCR to Baermann and KAPC increased the diagnostic yield in our study (Figs. 1 and 2), the sensitivity of the combination of Baermann and KAPC was not inferior to RTD-PCR (77.1% vs 74.3%; Table 2). Looking at other studies, the reported diagnostic performance of RTD-PCR varies across studies. While a study conducted in Côte d’Ivoire reports an inferior sensitivity of a combination of Baermann and KAPC compared to RTD-PCR (50.0% vs 76.8) [20], a study conducted in Cambodia reports that RTD-PCR shows a sensitivity of 88.9% when compared to a combination of Baermann and KAPC [21]. However, comparing results across studies is difficult, be it due to different hookworm coinfection rates, possibly leading to confusion of hookworm larvae with *Strongyloides* larvae, or due to other methodological differences. Especially at low intensity of infection, the considerably larger sample volume investigated by Baermann and KAPC compared to RTD-PCR may shift the sensitivity in favor for the conventional parasitological techniques. Since our patients were hospitalized due to other reasons, we speculate that the majority had most likely rather low *S. stercoralis* infection intensity.

Since *Strongyloides* larvae are discontinuously shed in feces, investigating more than one stool sample increases diagnostic sensitivity [5]. Although this approach is logistically more demanding, it allows increasing the diagnostic sensitivity even further than combining different diagnostic methods. In a study conducted in Cambodia the serial investigation of three stool samples for *S. stercoralis* by Baermann and KAPC increased the prevalence rate from 18.4% (1 sample) to 22.7% (2 samples) to 24.4% (3 samples) which was close to the mathematically modelled “true” prevalence rate of 24.8% [22].

Studies on *S. stercoralis* epidemiology from Southeast Asia indicate that *S. stercoralis* infection are predominately found in men, that the infection increases with age and that rural populations have a higher risk for infection [7, 8, 10, 14]. Our *S. stercoralis* patients follow these typical risk patterns although they have been recruited from a health services and not as the previous indicated studies from the community.

Our study would have benefited from a larger sample size. Although the major differences in yielding *S. stercoralis* diagnosis between the diagnostic techniques could be demonstrated, a more refined picture could have been drawn with a larger sample. Furthermore, including an analysis of several stool samples per patient would have further strengthened the validity of our results. However, already in about a third of our enrolled patients the provision of a stool sample was not possible. Increasing the stool collection would have further diminished patient compliance.

In summary, the imperfect sensitivity of all currently available diagnostic methods for detecting *S. stercoralis* in stool samples can be increased by parallel testing with different methods or by serial testing of several stool samples. The chosen strategy in daily clinical practice depends on the locally available resources and the desired certainty to detect or exclude *S. stercoralis* infection in an individual patient (e.g. diagnosing a patient with suggestive gastrointestinal symptoms [high infection intensity anticipated] vs screening of an asymptomatic patients before immunosuppressive therapy [low infection intensity anticipated]).

## Conclusions

A very high *S. stercoralis* prevalence of 33.7% was detected among in-patients of the Infectious Diseases Ward at Mahosot Hospital, Vientiane. We identified the combination of the Baermann technique and KAPC to be currently the most feasible and implementable standard for diagnosing *S. stercoralis* at Mahosot Hospital, and provincial and district hospital of Lao PDR and other low- and middle-income settings in Southeast Asia. Serial testing with this combination will be considered in cases where exclusion of *S. stercoralis* infection is of high clinical relevance (i.e. patients at risk for hyperinfection syndrome and disseminated strongyloidiasis).

## Data Availability

The dataset supporting the findings of this article is available from the corresponding author upon request.

## Abbreviations

CRS: Composite reference standard
DNA: Deoxyribonucleic acid
FECT: Formalin-ether concentration technique
KAPC: Koga Agar plate culture
KK: Kato-Katz technique
NPV: Negative predictive value
RTD-PCR: Real-time detection polymerase chain reaction
*CI*: Confidence interval
